# rSeqTU—A Machine-Learning Based R Package for Prediction of Bacterial Transcription Units

**DOI:** 10.3389/fgene.2019.00374

**Published:** 2019-05-15

**Authors:** Sheng-Yong Niu, Binqiang Liu, Qin Ma, Wen-Chi Chou

**Affiliations:** ^1^ Department of Computer Science and Engineering, University of California, San Diego, La Jolla, CA, United States; ^2^ School of Mathematics, Shandong University, Jinan, China; ^3^ Biomedical Informatics, College of Medicine, The Ohio State University, Columbus, OH, United States; ^4^ Infectious Disease and Microbiome Program, Broad Institute of MIT and Harvard, Cambridge, MA, United States

**Keywords:** machine learning, bacteria, transcription unit, R package, transcriptome

## Abstract

A transcription unit (TU) is composed of one or multiple adjacent genes on the same strand that are co-transcribed in mostly prokaryotes. Accurate identification of TUs is a crucial first step to delineate the transcriptional regulatory networks and elucidate the dynamic regulatory mechanisms encoded in various prokaryotic genomes. Many genomic features, for example, gene intergenic distance, and transcriptomic features including continuous and stable RNA-seq reads count signals, have been collected from a large amount of experimental data and integrated into classification techniques to computationally predict genome-wide TUs. Although some tools and web servers are able to predict TUs based on bacterial RNA-seq data and genome sequences, there is a need to have an improved machine learning prediction approach and a better comprehensive pipeline handling QC, TU prediction, and TU visualization. To enable users to efficiently perform TU identification on their local computers or high-performance clusters and provide a more accurate prediction, we develop an R package, named rSeqTU. rSeqTU uses a random forest algorithm to select essential features describing TUs and then uses support vector machine (SVM) to build TU prediction models. rSeqTU (available at https://s18692001.github.io/rSeqTU/) has six computational functionalities including read quality control, read mapping, training set generation, random forest-based feature selection, TU prediction, and TU visualization.

## Introduction

The gene expression and regulation in bacteria use different machinery from eukaryotic organisms. Operon has been defined as a set of genes controlled by a single promoter are first co-transcribed into one mRNA molecule, and then the mRNA molecule is translated into multiple proteins (Jacob et al., 1960). Operationally, an operon uses a single promoter to regulate the set of genes. Functionally, the set of genes in the operon encodes proteins with related biological functions. The *lac* operon in *Escherichia coli* is a typical operon that consists of a promoter, an operator, and three structural genes. The three genes, *lacZ*, *lacY*, and *lacA*, are co-transcribed into one mRNA transcript and are subsequently translated into three proteins, β-galactosidase, β-galactoside permease, and Galactoside acetyltransferase. The *lac* operon is responsible for the transport and metabolism of lactose in many enteric bacteria. The discovery of the *lac* operon won the Nobel Prize in Physiology by Jacob and Monod in 1965 (Jacob et al., 1960).

Recently, many works revealed bacterial genes are not transcribed only in single operons but may be dynamically co-transcribed into mRNAs with different gene sets under different growth environments or conditions (Yan et al., 2018). Each of the co-transcribed gene set is called transcription units (TUs). The concept of TU is analogical to alternative spliced protein isoforms in eukaryotic systems that use different exons to produce protein isoforms. Although alternative splicing can use nonadjacent exons, a TU consists of a set of adjacent genes.

Several operon databases, such as RegulonDB (Santos-Zavaleta et al., 2019), MicrobesOnline (Dehal et al., 2010), and ProOpDB (Taboada et al., 2012) provide various levels of operon information describing genes only expressed in single TU or operon. While DOOR2 (Mao et al., 2014) and OperomeDB (Chetal and Janga, 2015) provide the more comprehensive TUs describing genes are co-transcribed into different gene sets. Some TU or operon databases provide experiment-verified results while most of them rely on TU or operon predictions. Studies including DOOR2 (Mao et al., 2014), SeqTU (Chou et al., 2015), and Rockhopper (McClure et al., 2013) use genomic information and gene expression profile to predict operon or TU with machine learning and other approaches. Taboada et al. (2018) recently developed a new operon prediction method based on artificial neural network (ANN).

Other than *in silico* prediction works, Yan et al. recently used SMRT-Cappable-seq and PacBio sequencing to re-examine the transcription units of *E. coli* grown under different conditions to provide a higher resolution map of dynamic TUs (Yan et al., 2018). The work of Yan et al. revealed that TUs are better to describe the real bacterial transcription profiles and a gene can be contained in many different co-transcribed gene sets, TUs, under the same or different growth conditions. In our previous works (Chou et al., 2015; Chen et al., 2017), we assumed a gene can only be co-transcribed into only one adjacent gene set, which is one TU. We also assumed co-transcribed gene pairs follow transitive relation, and thus we connected co-transcribed gene pairs into a larger gene sets to form a TU.

In this study, we focused on improving our machine learning model for the prediction of the co-transcribed gene pairs and providing a user friendly R package, rSeqTU, for a comprehensive pipeline including RNA-seq read analysis, TU prediction, and TU visualization.

## Results

**Figure 1:**
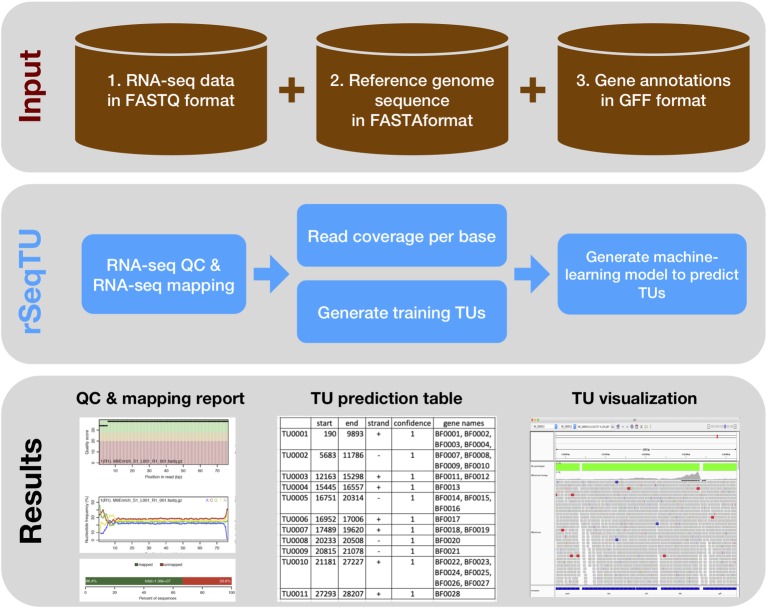
rSeqTU workflow uses input data to predict bacterial TUs. The rSeqTU workflow has three layers of schemas including input data, core processes, and major results. In the input data layer, rSeqTU needs RNA-seq data, reference genome sequence, and gene annotations. In the core process layer, rSeqTU performs QC, builds prediction models, and predicts TUs. The results layer includes the QC and mapping results, TU prediction tables, and files for visualization in IGV.

In this rSeqTU R package, we updated the TU prediction model with random forest-based feature selection and support vector machine (SVM). Besides, rSeqTU has a completed workflow performing RNA-seq read quality control (QC), RNA-seq read mapping, generation of TU results in two formats, and generation of IGV files for visualization (Figure 1).

rSeqTU requires three input data including RNA-seq data in FATSTQ format, reference genome sequence in FASTA format, and gene annotations in GFF format. With the input data, rSeqTU first performs RNA-seq data QC and RNA-seq read mapping to generate QC reports and mapping results in BAM format.

Then, rSeqTU uses whole genome per base read coverage and gene annotations to generate constructed TUs as the training data set. The constructed TUs are generated based on the SeqTU algorithm that was first presented by Chou et al. (2015). Briefly, the constructed TUs come from real single genes that are split into two adjacent sub genes with their intergenic regions to enable us to capture the continuity and stability features of RNA-seq signals of the real TUs. rSeqTU then applies random forest to select informative features using the constructed TUs and applies SVM to build a TU prediction model with the selected features.

rSeqTU reports the prediction accuracy and uses the TU prediction model to identify all co-transcribed gene pairs in the given genome. rSeqTU outputs TU prediction results in single gene pairs and concatenated gene pairs. Last, rSeqTU converts TU results into IGV-compatible files for TU visualizations. In short, rSeqTU produces RNA-seq read QC reports, RNA-seq mapping statistics and results, TU prediction results, and files for IGV visualization.

To evaluate rSeqTU R package, we used two sets of bacterial RNA-seq data of *Bacteroides fragilis* (*B. fragilis*) produced and published by Donaldson et al. (2018). These *B. fragilis* RNA-seq data were used to discover that human gut microbiome can use immunoglobulin A (IgA) to trigger robust host-microbial symbiosis for mucosal colonization. The study focused on investigating commensal colonization factors (CCFs), an operon, which was previously found to be essential for *B. fragilis* for colonization of colonic crypts (Lee et al., 2013). The CCF operon has five genes, *ccf*A-E, which are homologous to polysaccharide utilization systems, and the *ccf*A is activated by extracellular glycan sensing and is hypothesized to activate genes involved in mucosal colonization (Martens et al., 2009). To understand the function of *ccfA* gene, Donaldson et al. compared gene expression profiles between *ccfA* overexpressed *B. fragilis* and wild-type *B. fragilis* during laboratory culture growth. The RNA-seq data helped identify 24 out of 25 non-CCF genes that were differentially expressed and mapped to the biosynthesis loci for capsular polysaccharides A and C (PSA and PSC).

**Figure 2:**
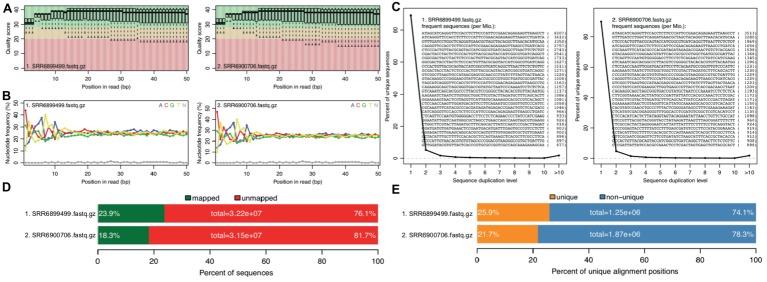
rSeqTU generates RNA-seq read QC reports and RNA-seq mapping statistics. SRR6899499 is *ccfA* overexpression data, and SRR6900706 is wild-type data. The panels **(A–E)** present quality scores, nucleotide frequency, sequence duplication, percentage of aligned bases plot, and percentage of unique and mapped reads.

With the two RNA-seq data sets, reference genome sequence, and gene annotations, we performed a full run of rSeqTU analysis. The RNA-seq data QC and RNA-seq mapping were generated and shown in Figure 2.

In Figure 2, we generated QC report for both *ccfA* overexpression and wild-type. It shows the read quality score plot, which is good in general over 30 (Figure 2A). Also, it generated nucleotide frequency plot (Figure 2B), sequence duplication plot (Figure 2C), percentage of aligned bases plot (Figure 2D), and percentage of unique and mapped reads (Figure 2E). We could observe that the sequence duplication is not severe. The nucleotide frequency, aligned bases, and mismatched bases information are in the normal range. The percentage of mapped reads and unique reads are lower than 30% as expected due to the most of the RNAs in the samples belong to mouse, the host, but not bacteria.

The two RNA-seq read mapping results were used to generate training data for TU prediction models, respectively. For *ccfA* overexpression data set, rSeqTU reported the sensitivity, specificity, and accuracy at 0.857, 0.999, and 0.963. For wild-type data set, rSeqTU reported the sensitivity, specificity, and accuracy at 0.885, 0.996, and 0.964. In general, we could find that rSeqTU generated high accuracy models after proper feature selections and cross-validation.

**Figure 3:**
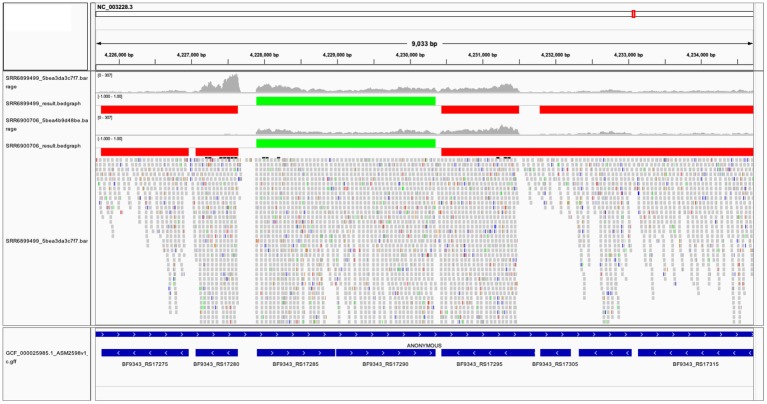
The visualization of predicted TUs on IGV. The predicted TUs are displayed in green and red bars for TUs on the forward and the reverse strands. The visualization also includes read coverage, gene annotations, and mapping results. SRR6899499 is *ccfA* overexpression data, and SRR6900706 is wild-type data.

The two TU prediction models were used to predict co-transcribed gene pairs. There are 1,759 and 1,626 co-transcribed gene pairs predicted in *ccfA* overexpression and wild-type RNA-seq data sets. If we concatenated co-transcribed gene pairs, rSeqTU identified 2,727 TUs including 2,079 single-gene TUs, 271 two-gene TUs, and 377 TUs with more than two genes in *ccfA* overexpression RNA-seq data set. In wild-type RNA-seq data set, rSeqTU identified 2,860 TUs including 2,249 single-gene TUs, 256 two-gene TUs, and 355 TUs with more than two genes. rSeqTU then uses the TU results to generate bedgraph files for the visualization in IGV (Figure 3). In Figure 3, we showed a region of *B. fragilis* genome containing eight genes. rSeqTU identified four TUs in the *ccfA* overexpression data (SRR6899499) and four TUs in the wild-type data (SRR6900706). However, the structure of the TUs is very different between two RNA-seq data sets. The two genes with locus tags, BF9343_RS17275 and BF9343_RS17280 were identified as a co-transcribed gene pair in the *ccfA* overexpression data but not in the wild-type data. The four genes with the locus tags, BF9343_RS17295, BF9343_RS17305, BF9343_RS17310, and BF9343_RS17315, were predicted as a single TU in the wild-type data but two TUs in the *ccfA* overexpression data.

To ensure the rSeqTU also performs well on RNA-seq data sets of different species, we took two RNA-seq data sets of uropathogenic *Escherichia coli strain* CFT073 to run TU predictions. The two data sets were used to investigate how *Escherichia coli strain* CFT073 senses and detoxifies nitric oxide (NO), which is a defense mechanism generated by host immune cells (Mehta et al., 2015). For without NO treatment RNA-seq data set, rSeqTU reported the sensitivity, specificity, and accuracy at 0.879, 0.997, and 0.952. For without NO treatment RNA-seq data set, rSeqTU reported the sensitivity, specificity, and accuracy at 0.824, 0.996, and 0.945.

## Materials and Methods

### New Functions Integrated or Invented by rSeqTU

rSeqTU uses QuasR R package to perform RNA-seq data QC and RNA-seq read mapping. The read mapping results are then processed by an algorithm named SeqTU first presented by Chou et al. (2015). In brief, the SeqTU algorithm splits relatively long single genes into three parts including two sub-gene regions and an intergenic region, and then SeqTU uses RNA-seq per-base read coverage over the three parts to generate TU features to describe the continuity and stability of RNA-seq read coverage. SeqTU assumes the RNA-seq read coverage within a TU is continuous and stable like it is within a gene.

rSeqTU selects essential TU features by random forest and builds TU prediction model by SVM using an R packages, Caret and e1071. rSeqTU converts TU prediction results into IGV-compatible files in bedgraph format for TU visualizations.

### Feature Selection by Random Forest

Random forest is a supervised learning algorithm using the ensemble learning based on decision trees. Random forest has been successfully used on biological data types such as genomics, transcriptomics, epigenomics, proteomics, and metabolomics (Degenhardt et al., 2019). rSeqTU uses recursive feature elimination to perform random forest and selects the top eight features. The top eight features may vary in different RNA-seq data sets, and the top few features are constantly fold change of adjacent gene expressions and proportion of gap positions in the whole given gene pair region.

#### *Bacteroides fragilis* RNA-seq Data

We used two RNA-seq data from each triplicate experiment from NCBI’s SRA database with project accession number PRJNA445716. The accession numbers of the two data sets are SRR6899499 (*ccfA* overexpression) and SRR6900706 (wild-type). The reference genome sequence and gene annotations of *Bacteroides fragilis* NCTC 9343 are GCF_000025985.1_ASM2598v1_genomic.fna and GCF_000025985.1_ASM2598v1_genomic.gff.

#### *Escherichia coli* RNA-seq Data

We used two RNA-seq data from each triplicate experiment from NCBI’s SRA database with project accession number PRJNA286883. The accession numbers of the two data sets are SRR2061823 (without NO treatment) and SRR2061826 (with NO treatment). The reference genome sequence and gene annotations of *Escherichia coli* strain CFT073 are GCF_000007445.1_ASM744v1_genomic.fna and GCF_000007445.1_ASM744v1_genomic.gff.

## Discussion

rSeqTU is a machine learning-based R package for TU prediction, empowered by a random forest algorithm for feature selection and multiple graphical visualizations and interactive tables for customized downstream analysis. Its superior prediction performance has been demonstrated by testing multiple RNA-Seq datasets in *B. fragilis*. The source code and tutorial of rSeqTU is available at https://s18692001.github.io/rSeqTU/.

rSeqTU will be useful to understand transcriptional profiles of bacterial genomes in the gene level and the TU level. In addition to the single bacterium, rSeqTU may also be applied onto the metatranscriptomic data, the RNA-seq data of microbiome. The TUs of multiple bacteria may provide systemic view to understand how microbiome regulates functional translation and can be integrated with other metagenomic and metabolomic data (Niu et al., 2018).

A TU is dynamically composed of different adjacent genes under various conditions, and different TUs may overlap with each other under the same and different conditions. The dynamic TUs sharing the same gene(s) are called alternative transcription units (ATUs), and the identification of ATUs is recognized as a more challenging computational problem due to their condition-dependent nature. Meanwhile, the third generation sequencing technology will shortly generate substantial genome scale ATU datasets in the public domain for various prokaryotic organisms. Hence, advanced computational models are urgently needed for ATU prediction based on RNA-Seq data.

Intuitively, the output of rSeqTU can lay a solid foundation of ATU prediction as (1) a TU identified in our method can represents a maximal ATU clusters with apparent promoter and terminator and (2) the TU can be used as an independent genomic region for further ATU prediction based on other genomic and transcriptomic features. If available, the ATUs along with related cis-regulatory motifs analysis will generate the dynamic regulatory networks in a bacterial genome to a higher resolution and an advanced level.

## Data Availability

Publicly available datasets were analyzed in this study. This data can be found here: https://www.ncbi.nlm.nih.gov/sra/?term=SRR6899499.

## Author Contributions

S-YN, QM, and W-CC designed the study. S-YN implemented the R package with W-CC’s help. S-YN, BL, QM, and W-CC wrote the manuscript.

### Conflict of Interest Statement

The authors declare that the research was conducted in the absence of any commercial or financial relationships that could be construed as a potential conflict of interest.

The handling editor declared a past co-authorship with one of the authors QM.
